# A Descriptive Website Review of Publicized PharmD Pre-Matriculation Programs

**DOI:** 10.3390/pharmacy14060131

**Published:** 2026-09-09

**Authors:** Colyn C. Bessard, Kala B. Oliver, Frank Yu, Ryoichi Fujiwara

**Affiliations:** 1 Department of Pharmaceutical Sciences and Health Outcomes, Ben and Maytee Fisch College of Pharmacy, The University of Texas at Tyler, Tyler, TX 75799, USA; cbessard@patriots.uttyler.edu (C.C.B.); koliver9@patriots.uttyler.edu (K.B.O.); 2 Department of Clinical Sciences, Ben and Maytee Fisch College of Pharmacy, The University of Texas at Tyler, Tyler, TX 75799, USA

**Keywords:** pharmacy, education, pre-matriculation, calculations, admission

## Abstract

**Objective:** Colleges of pharmacy have begun offering pre-matriculation programs to refresh and strengthen pharmacy students’ basic math skills. Although previous survey-based research showed that many institutions offer a pre-matriculation program, it remains unclear whether such information is publicly available to prospective pharmacy students. In this study, we conducted a comprehensive review of pharmacy school websites to identify pharmacy schools that publicly describe their pre-matriculation programs. **Methods:** The official websites of the Accreditation Council for Pharmacy Education pharmacy schools were searched for information on pre-matriculation programs. **Results:** Out of 142 pharmacy schools, 20 (14%) of those schools were found to describe their pre-matriculation program on their website. Ten of those 20 programs reported that their pre-matriculation program includes a section that teaches calculations. Only one school described a positive impact on student performance in a calculations course. The timing, length, and format of the 20 programs varied. **Conclusions:** Only a small proportion of U.S. pharmacy schools (14%) publicly disclose the presence of a pre-matriculation program on their institutional websites. Most of the programs do not report outcomes or evidence of impact. Future work should focus on developing calculation-focused pre-matriculation programs and evaluating their effectiveness, while also improving the visibility and availability of information about these programs on institutional websites.

## 1. Introduction

The Doctor of Pharmacy (PharmD) degree in the United States is a professional program designed to prepare graduates to participate in interprofessional collaborations, medication management, and clinical decision-making [1]. Early in the PharmD curriculum, student pharmacists begin to obtain knowledge from courses such as pharmacology, pharmaceutics, and most importantly, pharmaceutical calculations. Mastering pharmaceutical calculations early on in a student’s career is the key to succeeding in future coursework such as pharmacokinetics, sterile and non-sterile compounding, and even experiential education [1]. Not only is the pharmaceutical calculations course a fundamental component of pharmacy practice, but it is essential for the safe and accurate preparation, dispensing, and administration of medications. Pharmacists routinely apply calculation skills when determining medication doses, concentrations, infusion rates, and quantities. This makes mathematical competency directly relevant to patient safety [2]. Errors in these calculations can contribute to medication errors and potentially result in patient harm. Therefore, developing strong pharmaceutical calculation skills during pharmacy education is essential to preparing student pharmacists for safe and effective clinical practice.

Furthermore, competency in pharmaceutical calculations is also a requirement to become a licensed pharmacist. The National Association of Boards of Pharmacy (NABP) administers the North American Pharmacist Licensure Examination (NAPLEX) to those who have earned their PharmD degree. Previously, NAPLEX content followed the 2021 Competency Statement blueprint, which included six domains. One of those domains was dedicated entirely to performing calculations and made up 14% of the exam’s content. Due to a blueprint revision, exams taken after 1 May 2025 followed the updated 2025 NAPLEX Content Outline, which now included only five domains. With this update, pharmaceutical calculations content is now integrated into a broader domain titled Foundational Knowledge for Pharmacy Practice, which makes up 25% of the exam. In addition to testing calculation skills, this domain also includes questions regarding pharmaceutical sciences, compounding, drug development, research design, biostatistics, and drug information resources [3].

Although we recognize the importance of calculation competency in the field of pharmacy, we also recognize that pharmaceutical calculations remain a common area of difficulty for many pharmacy students. In 2011–2013, the mean NAPLEX passing rate for pharmacy schools in the United States was 96.2%, but in 2021–2023, this percentage dropped to 78.7%, which is potentially attributed to poor pharmaceutical calculation performance [4]. This conclusion is supported by an observation that approximately 30% of pharmacy students earned a level 1 or 2 in the Foundational Knowledge for Pharmacy Practice domain, which includes pharmaceutical calculations concepts [5]. A score of 1 or 2 denotes that the student did not master the concepts tested in that section. To show competency in a subject, a student should score a 3 or 4. Mastering pharmaceutical calculations is not only integral to passing the NAPLEX exam, but it also prevents medication errors from occurring in the field of pharmacy. In healthcare settings, calculation mistakes still contribute to many medication errors, like dosing inaccuracies, ultimately causing patient harm. To proactively mitigate this gap in mathematical skills, most colleges of pharmacy have placed their pharmaceutical calculations course in the first professional year. This early introduction allows students time to establish accuracy, efficiency, and confidence in performing life-saving calculations, but introducing calculations early in the PharmD curriculum is not the complete solution.

One potential reason students are struggling with calculations is that pharmacy students are already coming in with a knowledge deficit in math. When mathematics pretests were given to incoming pharmacy students at the University of South Florida and Virginia Commonwealth University, the mean test score of the 922 students was 73.8%, but overall performance differed between the two schools. Not only are students showing themselves to be unprepared in the calculations department, but another problem was displayed, which is the variance in math proficiency among students [6]. When it comes to students applying to pharmacy schools, there is an array of backgrounds that are being accounted for. Some students have a prior degree, some only completed the required prerequisites if that was an option, and some were already established in a different career for several years before they applied to pharmacy school. The time that has elapsed between each student’s most recent math class and their start of pharmacy school’s calculations courses also influences their math ability. One study found that students who have a longer time period between completing their last schooling and starting pharmacy school had lower grades in both their PharmD calculations and kinetics courses. Also, students who took more math courses in high school or college, prior to entering pharmacy school, had better grades in their PharmD calculations course [7]. Contributing factors may include variability in students’ prior mathematical preparation, such as differing K-12 curricula, differences in prerequisite coursework requirements, and the discontinuation of standardized assessments such as the Pharmacy College Admission Test. As there is a notable decline in pharmaceutical calculations performance, understanding the underlying causes is essential to addressing this educational challenge. Crawford et al. describe declining performance in pharmaceutical calculations as multifactorial, with contributing factors related to students’ secondary and undergraduate educational backgrounds [5]. Students entering PharmD programs come from diverse educational pathways, which can influence baseline quantitative skills and preparedness for calculation-based coursework. At the secondary education level, the Common Core State Standards (CCSS), implemented in 2010, aimed to standardize mathematics instruction and influence the foundational quantitative skills students bring into undergraduate education; however, their impact on pharmaceutical calculation performance remains unclear. Analysis of National Assessment of Educational Progress (NAEP) data demonstrated declines in mathematics performance among fourth- and eighth-grade students between 2013 and 2015, interrupting approximately 25 years of small-to-moderate gains in mathematics achievement [8]. Additionally, states adopting the CCSS did not demonstrate greater improvements in mathematics or reading achievement than non-CCSS states between 2003 and 2015 [9]. The subsequent passage of the Every Student Succeeds Act in 2015 shifted greater responsibility for educational standards and accountability to individual states, while national assessments continued to identify concerns regarding mathematics performance [10]. Unlike secondary education, undergraduate coursework is not guided by national curricular standards and instead varies by institution and program, contributing to heterogeneity in students’ foundational mathematical training prior to PharmD admission [11]. Supporting this variability, prior experiences with college-level mathematics and confidence in mathematical ability have been shown to be associated with performance in pharmaceutical calculations coursework, underscoring the importance of undergraduate quantitative preparation [7]. In addition, changes to prerequisite coursework requirements across pharmacy programs may further contribute to variability in students’ exposure to quantitative and problem-solving content [12]. For example, approximately 9% of PharmD programs have removed physics from their prerequisite requirements, illustrating differences in required mathematics-based coursework prior to admission [11]. Although physics is not a direct measure of pharmaceutical calculations proficiency, it represents an applied quantitative discipline that reinforces mathematical reasoning skills relevant to pharmacy education. Given the identification of these contributing factors, colleges of pharmacy and the academy have been encouraged to explore strategies to address gaps in calculation preparedness, including prerequisite modifications and the implementation of pre-matriculation programs prior to admission [5].

Pharmacy programs have been encouraged to explore strategies to address gaps in calculation preparedness. As a result, some programs have implemented pre-matriculation programs. For example, one school of pharmacy implemented a 5-week program during the summer before P1 classes began and compared the GPAs and first-year probation risks of students who participated in the summer course versus those students who decided not to participate. Although this was limited to a single institution, they found that non-participants had a 5.24 times higher risk of first-year probation, as compared to those students who did participate [13]. A pre-matriculation program is an educational bootcamp that is designed to strengthen foundational knowledge, facilitate academic success, and prepare accepted students for the rigorous first-year course load before they officially begin classes. Some pharmacy schools make their pre-matriculation programs mandatory, while other pharmacy schools allow attendance to their pre-matriculation programs to be optional [14]. These programs typically provide instruction on mathematics, biomedical sciences, writing, professionalism, and study skills. While these programs aim to increase confidence, retention, and academic performance, reported outcomes have been mixed due to variations in program structure, timing, duration, delivery methods, curriculum depth, and topics [15]. When 120 pharmacy programs across the United States were surveyed, 46 schools self-reported offering a pre-matriculation program. Among these 46 schools, the effectiveness of their pre-matriculation program, measured by on-time graduation rates, was reported as positive by 10%, somewhat positive by 45%, and neutral by 48% [16]. The survey results suggest that many schools either do not perceive their program as highly effective or are not currently able to offer one due to limited available resources such as housing or staffing. One critique of this study is that because it was a distributed survey, a school that discloses that it offers a pre-matriculation program does not always coincide with that school describing the pre-matriculation program on its website. Due to this discrepancy, we chose to look at pharmacy schools’ websites to identify the amount of available public information on pre-matriculation programs. We believe that providing public information on whether a school has a pre-matriculation program is good practice, offering transparency to students. Pre-matriculation programs may be seen as positive to students who recognize the possible benefit, but contrarily, some students who are strong academically may not find a perceived benefit in completing an additional program before starting their curriculum.

This study aims to compile a comprehensive list of accredited pharmacy programs in the United States that publicly describe their pre-matriculation program on their official school website. We will also evaluate each pre-matriculation program’s characteristics, including length, format, topics, and reported outcomes, with an emphasis on the inclusion of pharmaceutical calculation material. By organizing the available data on these programs, this study aims to identify how many pharmacy programs have a pre-matriculation program, which schools describe them on their websites, and to what extent pharmaceutical calculations are integrated into the program. The focus of this study was to identify which of those schools designed a program to decrease the gap in calculation-based knowledge before their newly admitted students began their first semester of courses.

## 2. Methods

This descriptive website review was completed between January and July 2026 and was designed to compile data on accredited pharmacy school programs in the United States. The list of schools included in the study was taken from the Accreditation Council for Pharmacy Education (ACPE) website and included a total of 142 schools. Inclusion criteria included ACPE pharmacy schools that offered programs that incorporated preparatory work with a focus on calculations or remediation-like modules for their recently accepted students to complete the summer before they matriculate. Exclusion criteria included programs that were tailored towards career exploration, aimed towards high school or undergraduate students, concurrently taken with courses during the semester, and programs that did not mention a curriculum or calculations-focused component.

To organize the extracted information, a spreadsheet was utilized to collect variables such as state, dates, length, format, presence of a calculations component, and any reported outcomes. Reported outcomes were defined as any statement describing the impact of the pre-matriculation program on student academic performance that was publicly available on the institution’s website, including findings from published studies and unpublished institutional data. To begin the data collection, two primary research investigators split the number of schools on the ACPE website evenly, ensuring that each pharmacy school’s website was thoroughly reviewed by one investigator. The pharmacy schools’ websites were investigated by searching for the terms prematriculation/pre-matriculation, primer, bridge, prep, readiness, summer, and orientation exclusively. The term “calculations” or “pharmaceutical calculations” was removed from our list of search terms after we noticed that searches were not fruitful just using that term alone. If searching one of those terms resulted in finding information on the school’s pre-matriculation program, no further investigation was necessary.

## 3. Results

An extensive search of institutional websites identified 20 of the 142 U.S. schools and colleges of pharmacy (14.1%) that publicly described a pre-matriculation program (Table 1). Of these 20 institutions, 10 (50%) reported including a pharmaceutical calculations component in their pre-matriculation program. In addition to pharmaceutical calculations, frequently reported curricular topics included study skills, biomedical sciences, medical terminology, physiology, biochemistry, professionalism, and academic success strategies. Seven institutions (35%) reported a positive academic impact associated with their pre-matriculation program. Reported outcomes ranged from retrospective comparisons demonstrating improvements in course performance between cohorts to student testimonials describing perceived benefits following program completion. Among the 10 institutions that included a pharmaceutical calculations component, five reported generalized positive academic outcomes across multiple subject areas, whereas only one institution specifically evaluated the impact of its pharmaceutical calculations module. That institution, S15 on Table 2, reported a significant improvement in student performance in the first-year pharmacy calculations course following implementation of its pre-matriculation pharmaceutical calculations module in two separate studies done in 2023 and 2026 [17,18].

An area of interest was the timing of the pre-matriculation program, which varied across all institutions. From the 20 schools, seven schools were found to have their program in August or right before the fall semester, 10 institutions reported having their program take place during the summer (June–July), and three schools did not report when their program takes place (Table 3). Another area of interest was the length of the program. A total of five schools (25%) reported that their pre-matriculation program lasted between 1 and 7 days, two institutions (10%) reported their program lasted 8–14 days, and 6 institutions (30%) lasted 15+ days, with the longest reported program lasting five weeks. The remaining institutions were either self-paced (5 of 20) or unknown (2 of 20). The last area of interest was the format of the pre-matriculation program. Of the reported schools, seven (35%) institutions reported in-person, 10 (50%) reported virtual, two (10%) reported hybrid, and one (5%) was unknown.

## 4. Discussion

There is no standardization of pre-matriculation programs at pharmacy schools across the United States. Some pharmacy schools do not seem to offer any form of a pre-matriculation program, while other schools that do offer them still experience gaps in the program’s design, specifically relating to the integration of pharmaceutical calculations content and positive student outcomes. When prospective students are deciding which pharmacy program to attend, there are several variables that are commonly considered, as seen in Table 4. Students are known to select their program of choice based on location, financial obligations, familiarity, institutional characteristics, and personal academic statistics [19,20]. When first-year pharmacy students from several universities were asked what factors influenced their decision when choosing a school, the most common answers were attributed to the school’s image [19]. Following, other answers included safety, program offerings, and finances [19]. A second study interviewed first-year students at two other colleges of pharmacy. These students noted that their motivating factors included institutional characteristics, financial variables, influence from others, and the admissions process [20]. Unsurprisingly, none of the interviewees noted that a school’s pre-matriculation program swayed their decision one way or the other. One possible explanation of this is that prospective students are not presented with information on pre-matriculation programs when they are doing their research on schools. The pharmacy school a person attends is a very important decision that can affect the future of their academic success and career pathways. The discrepancy between how many schools claim to offer a pre-matriculation program and how many pre-matriculation programs are found when searching school websites should be discussed more. Comparing two different survey studies showed that within a time span of three years, there was an increase in schools that self-reported offering a pre-matriculation program [16,21], but this increase was not apparent through our website research (Table 1). The study done by Klausner et al. in 2016 yielded results showing that out of the 50 pharmacy schools that participated in their survey, 49 of them conducted orientations, 5 conducted boot camps, and 6 conducted pre-matriculation programs [21]. Sessions dedicated to math or calculations appeared in less than 4 of these programs. The study done by the American Association of Colleges of Pharmacy Student Affairs Committee in 2023 showed that out of the 120 schools that participated in their survey, 46 of them offered a pre-matriculation program [16]. When we conducted our website review, we only found information on 20 schools that disclosed that they offered a pre-matriculation program. The fact that not many pharmacy schools are marketing their pre-matriculation programs to prospective students on their school websites gives us reason to infer that pharmacy schools may presume that publicly reporting a pre-matriculation program’s information on their school website might increase the risk of deterring students from applying or wanting to attend. But one suggestion for pharmacy schools with that belief is to put effort into designing an exemplary pre-matriculation program that yields positive student outcomes. If a school has a mediocre pre-matriculation program, or one that does not prove to offer any additional benefit, then a student, whether academically strong or academically weaker, would likely not be interested in participating. But if the program’s outcomes are positive, it may be in the school’s interest to market their pre-matriculation program appealingly. Going forward, all schools that offer a pre-matriculation program should list the program on their website and describe it in acceptable detail.

Beyond the visibility and overall design of pre-matriculation programs, pharmacy schools should also consider the specific academic challenges these programs are intended to address, including mathematics anxiety. Mathematics anxiety represents an important, yet often underrecognized, factor that may influence student success in pharmacy education.

Mathematics anxiety has been described as feelings of tension, apprehension, or fear that interfere with mathematical performance and can negatively affect learning, confidence, and academic achievement [22,23]. Students experiencing mathematics anxiety are more likely to avoid quantitative tasks, demonstrate lower self-efficacy, and devote cognitive resources to anxiety rather than problem-solving, ultimately impairing mathematical performance [23]. Within the health professions, these concerns are particularly relevant because medication dosage calculations require both mathematical competency and accuracy. A scoping review by Williams et al. found that mathematics anxiety was consistently associated with medication dosage calculation performance among nursing students and identified a need for additional research examining these relationships in other healthcare disciplines, including pharmacy [12]. The findings of our study suggest that pharmaceutical calculations remain an underutilized component of pharmacy pre-matriculation programs (Table 1). Although pharmaceutical calculations are considered a foundational competency for pharmacy students, only half of the identified programs explicitly incorporated a pharmaceutical calculations component, and only one institution specifically evaluated its impact on first-year pharmaceutical calculations performance (Table 1). Educational interventions that strengthen foundational mathematical knowledge and improve students’ confidence before entering professional coursework may help reduce mathematics anxiety and enhance academic preparedness [23]. While our current study did not evaluate changes in mathematics anxiety, incorporating structured pharmaceutical calculations into pre-matriculation programs may provide students with an opportunity to review essential concepts in a supportive, low-stakes learning environment before encountering the rigor of the PharmD curriculum. This early exposure may help reduce mathematics anxiety as students progress through the program.

Students who recognize mathematics as an area of concern may be more likely to seek institutions that visibly offer structured academic support prior to matriculation. Conversely, students with mathematics anxiety may be less inclined to apply to schools that prominently emphasize pharmaceutical calculations. Our study did not examine the influence of publicly available program information on school selection. Future research should examine whether the availability and visibility of these programs influence applicant decision-making, program participation, mathematics self-efficacy, and subsequent academic performance.

Another important consideration identified in our study is the timing and accessibility of pre-matriculation programs. Half of the identified programs were offered during the summer months (June–July), whereas over one-third were conducted immediately before the start of the fall semester (Table 3). Although current evidence does not indicate that one approach is superior, each timing strategy may offer distinct advantages. Programs offered earlier in the summer may provide students with additional time to review foundational concepts, strengthen mathematical skills, and address knowledge gaps before beginning the PharmD curriculum. In contrast, programs conducted immediately before matriculation may minimize knowledge decay and allow students to transition directly into professional coursework. Program timing should also be considered alongside student availability, as many incoming pharmacy students balance employment, relocation, family responsibilities, or other commitments during the summer before pharmacy school. Offering flexible formats, such as self-paced or hybrid programs, may improve accessibility while allowing students to engage with preparatory material at their own pace. Future research should evaluate whether program timing and flexibility influence participation, academic preparedness, knowledge retention, and student performance.

One of the limitations of this study is that although we made strong efforts to identify all the publicly reported information on pharmacy schools’ pre-matriculation programs from their websites, there is a possibility that some information that is available was missed. Though we followed the search protocol outlined in our methods section, we noticed that the information was not found in the same location when comparing each school’s data. Some schools mentioned pre-matriculation programs in the Student Success section of their website, whereas others included them under Accepted Students or Student Resources. We also want to point out that we are aware that publishing information on the school’s public website is not the only way for the school to communicate with its prospective or incoming students. It is possible that schools may decide to disclose information regarding their pre-matriculation programs through other means of communication, such as social media, emails, or student portals, just to name a few. On the other hand, pharmacy programs may intentionally avoid disclosing information regarding their pre-matriculation program offerings. This option may be utilized by schools that do not make their pre-matriculation program mandatory or schools that collaborate with other departments or colleges to host such a program. Since we did not evaluate any other means of communication other than school websites, we would like to identify this as a limitation to our study. Consistency in where all schools upload this type of information would allow prospective students a single place to look when doing their research on schools, as well as allow for more reliability and assurance in future comprehensive search studies similar to this one. One study found that when it comes to first-generation students who need more access to support services, it is integral to ensure that the information can be easily discovered, preferably within a few clicks on their college’s website. They went on to say that the desire to support these students can be optimized by communicating through their school’s website, so students are made aware that these resources exist and are available to them [24]. The same can be said for our research topic: that entering pharmacy students need more academic support in pharmaceutical calculations, and if that support is provided to students through a pre-matriculation program, then the program should be easily found on the school’s website within a few clicks.

Another limitation of the current study was that the depth and quality of the pharmaceutical calculations instruction within the described pre-matriculation programs were not evaluated and could not be evaluated due to the nature of the study. Data extraction was limited to information publicly available on the pharmacy institutions’ websites, which met the inclusion criteria if a program included a calculations program, but this provided little detail regarding actual instructional content, teaching strategies, the amount of time devoted to just calculations, or any assessment methods that could have been included. Because of this, programs that listed pharmaceutical calculations as part of the material were all categorized into one group despite potential differences that could not be identified. Future research should not only include the presence of pharmaceutical calculations in a pre-matriculation program, but also how they are taught and how differences in instructional depth influence students’ academic performance.

## 5. Conclusions

This study provided a comprehensive overview of pharmacy pre-matriculation programs described by schools and colleges of pharmacy within the United States, with an emphasis on the inclusion of pharmaceutical calculations. One of the key findings was that only 14% of pharmacy schools publicly described their pre-matriculation programs, and only half of those programs included pharmaceutical calculations as a component. Furthermore, few institutions reported academic outcomes, and only one specifically evaluated the impact of the pharmaceutical calculations component on student performance. Given the potential role of pre-matriculation programs in supporting incoming students and communicating institutional resources, future research should investigate whether the visibility and accessibility of publicly available pre-matriculation program information influence prospective student engagement, application decisions, and pharmacy school enrollment. Additionally, studies should evaluate the effectiveness of these programs using standardized outcome measures, examine the influence of instructional design and program timing, and determine how pharmaceutical calculations instruction affects student confidence, academic performance, and long-term success. Continued development and rigorous evaluation of pre-matriculation programs may help identify best practices for supporting incoming pharmacy students and promoting a successful transition into professional education.

## Figures and Tables

**Table 1:** Characteristics and Reported Outcomes of Publicly Described Pharmacy Pre-Matriculation Programs in the United States.

Outcome Measure	Findings
Pharmacy schools with publicly described pre-matriculation programs	20 of 142 schools (14%)
Pre-matriculation programs including pharmaceutical calculations instruction	10 of 20 programs (50%)
Pre-matriculation programs reporting positive academic outcomes	7 of 20 programs (35%)
Pre-matriculation programs including calculations that reported positive outcomes specifically related to calculations	1 of 10 programs (10%)

**Table 2:** Overview of Pre-Matriculation Programs at U.S. Colleges of Pharmacy.

School/College of Pharmacy Name	Timing	Length	Calculation Included?	Format	Topics Covered in Pre-Matriculation Programs	Positive Outcome Published?
School #1 (S1)	August	7 days	No	Hybrid	Role of the pharmacist in healthcare, including team member, interprofessional practice, and continual professional development	No
School #2 (S2)	July	6 weeks	Yes	Virtual	Professional development, clinical content, orientation to graduate school/PharmD rigor, academic success, wellness, and team readiness	No
School #3 (S3)	Unknown	2 weeks	Yes	Unknown	Sciences, math, top 200 drugs, and study skills	No
School #4 (S4)	July	4 weeks	Yes	Hybrid	Math, biochemistry, and student success strategies	No
School #5 (S5)	August	N/A	No	Virtual	Anatomy, statistics, foundational knowledge for PharmD success	No
School #6 (S6)	August	2 days	Yes	Hybrid	Sciences, math, medical terminology, and study skills	No
School #7 (S7)	August	N/A	No	Virtual	Navigating canvas, time management, netiquette, student success	Yes
School #8 (S8)	July	5 weeks	No	In person	Academic preparation, and mentoring	No
School #9 (S9)	July	4 weeks	Yes	In person	Sciences, math, and top 300 drugs	Yes
School #10 (S10)	June-August	Self-paced	No	Virtual	Biochemistry, genomics, physical and pharmaceutical chemistry, abilities lab, general patient management	Yes
School #11 (S11)	August	2 days	No	In person	Discussions about evidence-based learning, curricular demands	No
School #12 (S12)	Unknown	Self-paced	No	Virtual	Anatomy, physiology, molecular biology, and microbiology	No
School #13 (S13)	Unknown	Self-paced	Yes	Virtual	Course expectations, introduction to curriculum, resources and support, professionalism	No
School #14 (S14)	June	4 weeks	Yes	In person	Sciences, math, and pharmacy practice	Yes
School #15 (S15)	Unknown	Self-paced	Yes	Virtual	Pharmaceutical Calculations	Yes
School #16 (S16)	Unknown	self-paced	No	Virtual	Program-specific subject matter, learning and test-taking skills, tips for success, and library resources	No
School #17 (S17)	August	2 weeks	No	In person	Unspecified	No
School #18 (S18)	June	4 weeks	Yes	Virtual	Sciences, math, and medical terminology	Yes
School #19 (S19)	August	7 days	No	In person	Preparatory orientation	No
School #20 (S20)	July	4 days	Yes	Virtual	Sciences and math	Yes

**Table 3:** Characteristics of Publicly Described Pharmacy Pre-Matriculation Programs.

Characteristic	Category	n
Timing	Summer (June–July)	10
	Immediately before the fall semester (August)	7
	Unknown	3
Length	1–7 days	5
	8–14 days	2
	≥15 days	6
	Self-paced	5
	Unknown	2
Format	Virtual	10
	In-person	7
	Hybrid	2
	Unknown	1

**Table 4:** Factors that Influence Pharmacy School Choice.

Category	Factors
Student characteristics	Background Demographics Socioeconomic status Personal academic statistics (test scores, GPA, PCAT)
Personal and social influences	Familiarity with the institution Influence from others (peers, faculty, friends, family)
Academic experience	Program offerings Extracurricular experiences
Institutional characteristics	School image (reputation, ranking) Campus attributes (size, GPA requirements, Carnegie classification) Location (proximity to family, safety)
Financial considerations	Tuition Financial aid Scholarships Loans
Recruitment and admissions	Marketing by the university Admissions process (application, communication, interview)

## Data Availability

The original contributions presented in this study are included in the article. Further inquiries can be directed to the corresponding author.
